# Delusional disorder and tuberculosis: A clinical case

**DOI:** 10.1192/j.eurpsy.2021.1718

**Published:** 2021-08-13

**Authors:** V. Podence Falcão, R. Avelar, C. Abreu, M. Heitor

**Affiliations:** 1 Psychiatry, Hospital Beatriz Ãngelo, Loures, Portugal; 2 Internal Medicine, Hospital Beatriz Ãngelo, Loures, Portugal

**Keywords:** mental health, psychosis, tuberculosis

## Abstract

**Introduction:**

Tuberculosis is still a challenging disease, infecting around a third of the world’s population. As comorbidity with mental disorder is common, it is relevant to associate them at a diagnostic, therapeutic and prognostic level.

**Objectives:**

We present a clinical case describing a patient with psychosis, further diagnosed with tuberculosis during psychiatric treatment. Moreover, we present a summarized revision of the state of the art.

**Methods:**

Revision of the state of the art, drawing from PubMed and using the keywords “mental health”, “psychosis” and “tuberculosis”, in the last 10 years.

**Results:**

Male, 61 years old, heavy smoker and alcohol drinker. Admitted for allegedly feeling “worms” in his body. After medical examination, a weight loss of 13 kg in five months and symptoms compatible with tenesmus stood out. Following diagnostic tests, the patient was diagnosed with Ekbom Syndrome and Ganglionar Tuberculosis; he was then medicated with the adequate antipsychotic and tuberculostatic agents, which resulted in overall clinical improvement.

**Conclusions:**

This case illustrates the relationship between tuberculosis and mental disorders, in a patient with a low literacy level and a precarious socioeconomic background, known risk factors for mental disorder in patients with tuberculosis and are often associated with poor therapeutic adherence. Although proper treatment of the mental disorder is key to reducing the risk of tuberculostatic dropout, the stigma of mental disorder and tuberculosis decreases the probability of these patients seeking proper treatment. Thus, we alert the medical community for the possibility of psychiatric comorbidity in patients with diagnosed tuberculosis – and vice-versa –, allowing for an early intervention,

**Disclosure:**

No significant relationships.

